# Superiority of the Bag-Valve-Guedel Adaptor Over the Standard Face Mask for Preintubation Ventilation of Bearded Patients by Trainees With Limited Experience: Prospective Controlled Cross-Over Clinical Trial

**DOI:** 10.1016/j.acepjo.2024.100035

**Published:** 2025-01-13

**Authors:** Lilach Gavish, Shimon Firman, Daniel Fernando Orjuela Cruz, Anat Tovim, S. David Gertz, Roger Andres Gomez Barrantes, Dina Velitsky, Angelika Erport, Joel Shapiro, Chloe Mimouni, Arik Eisenkraft, Reuven Pizov

**Affiliations:** 1 Institute for Research in Military Medicine, Faculty of Medicine, The Hebrew University of Jerusalem, Israel Defense Forces Medical Corps, Jerusalem, Israel; 2 Department of Medical Neurobiology, The Saul and Joyce Brandman Hub for Cardiovascular Research, Institute for Medical Research, Faculty of Medicine, The Hebrew University of Jerusalem, Jerusalem, Israel; 3 Department of Anesthesiology, Critical Care and Pain Medicine, Hadassah – Hebrew University Medical Center Jerusalem, Jerusalem, Israel; 4 Department of Intensive Care, Christchurch Hospital, Christchurch, New Zealand; 5 Department of Anesthesiology and Pain Medicine, Canton Hospital of Fribourg, Fribourg, Switzerland

**Keywords:** airway management, ventilation of the bearded, preintubation ventilation, difficult mask ventilation, resuscitation

## Abstract

**Objectives:**

Ventilation of bearded patients using the standard face mask (FM) is often difficult, particularly in field settings and mass casualty events. The current study compares the effectiveness of a novel Bag-Valve-Guedel Adaptor (BVGA) with the standard FM when applied to anesthetized patients by anesthesiology trainees with limited experience.

**Methods:**

Male patients scheduled for elective surgery (American Society of Anesthesiology physical score 1-2) were recruited for this prospective, randomized, cross-over trial. Beard length was categorized as <0.5 cm (none/stubble), 0.5 to 1 cm, 1 to 5 cm, or ≥5 cm. Anesthetized patients were ventilated by anesthesiology trainees using the BVGA and an FM. The main outcome included end-tidal CO_2_, expiratory tidal volume (tidal volume of predicted body weight), and user evaluation (comfort, physical demand, and tiredness). The role of the level of expertise was evaluated by comparing data from the present study with those of a previous study performed by attending anesthesiologists.

**Results:**

Forty men (mean ± SD, age, 37 ± 17 years; body mass index, 25 ± 3 kg/m^2^), of whom 30 had beards, were enrolled. For the BVGA, ventilation parameters were found to be superior to the FM (BVGA vs FM: end-tidal CO_2_ [mm Hg], 34.3 ± 4.9 vs 26.6 ± 5.8, *P* < .001; expiratory tidal volume [mL/kg predicted body weight], 7.9 ± 2.5 vs 6.3 ± 2.8, *P* = .003). The BVGA was graded as more comfortable and less physically demanding by 96% to 100% of trainees. The level of expertise of the anesthesiologist (trainee vs attending [additional n = 61 patients]) and the presence of a beard were found to be significant factors for ventilation with the FM but not with the BVGA.

**Conclusion:**

The BVGA provides more effective and convenient ventilation than the FM for ventilation even when applied by anesthesia trainees. Its use can be of particular value in bearded subjects or in a setting where the use of supraglottic airway devices is limited.

**The Bottom Line**

Having a beard is a leading, independent predictor of difficult or impossible mask ventilation because of interference with the seal between the mask and face. The Bag-Valve-Guedel Adaptor is an intraoral device that provides a direct connection between a manual resuscitator and a Guedel oropharyngeal airway. We show that compared with the conventional face mask, the Bag-Valve-Guedel Adaptor provides superior respiratory parameters (end-tidal CO_2_ [mm Hg], mean ± SD: 34.3 ± 4.9 vs 26.6 ± 5.8, *P* < .001) and more convenient ventilation, particularly for bearded subjects, even when administered by anesthesiology trainees.

## 1 Introduction

### 1.1 Background

Recent surveys indicate that 55% of men globally sport a beard – 52% in the UK and 33% in the US.^1^, ^2^, ^3^ Nonetheless, having a beard has been identified as a leading, independent predictor of difficult or impossible mask ventilation because it interferes with the seal between the mask and face.^4^, ^5^, ^6^, ^7^ Suggested solutions for preintubation ventilation of bearded patients have included shaving off the beard, covering the face with a transparent medical dressing, or applying gel on the edge of the mask.^8^, ^9^, ^10^, ^11^ None of these is easily applied during emergency situations. The supraglottic laryngeal mask airway (LMA) is frequently used in such situations. However, an intraoral airway is less invasive and less irritating, requires a lesser depth of unconsciousness, and avoids other known possible complications of the LMA.^12^

### 1.2 Importance

**Figure 1:**
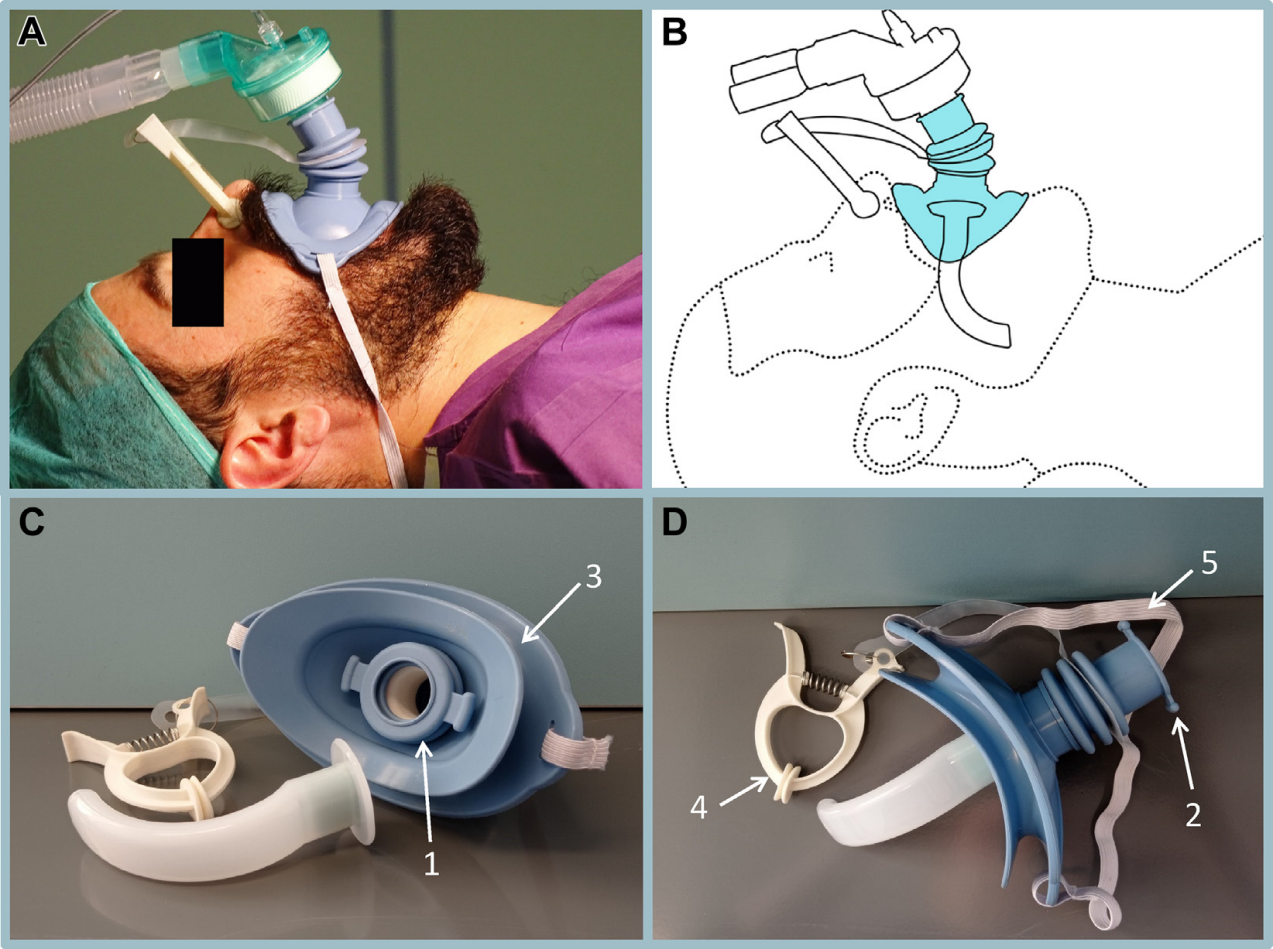
Bag-Valve-Guedel Adaptor (BVGA). Components of the BVGA include (1) a universal adaptor on the internal side for the Guedel airway (AW), (2) a universal adaptor on the external side for the ventilation bag, (3) a mouth seal element, (4) a nose seal element, and (5) a wide rubber band to secure the BVGA in place. The lips fit into the BVGA mouth seal element, and the nose seal element completes the seal, permitting the direct flow of air from the bag valve to the airway. (A) BVGA in situ applied to a bearded patient. (B) Schematic of the BVGA with an AW. (C) Inferior aspect of the BVGA. (D) BVGA is connected to an AW and ready for insertion.

In order to circumvent the difficulty in ventilating bearded patients, we tested an intraoral Bag-Valve-Guedel Adaptor (BVGA) that provides a direct connection between a manual resuscitator and a Guedel oropharyngeal airway without the need for a face mask (FM) (Fig 1).^13^ The BVGA, constructed from medical-grade silicone, has a mouth seal element and universal adaptors for the Guedel airway and the ventilation bag, as well as a separate nose seal element (Fig 1). A wide rubber band secures the BVGA to the face. We showed that the BVGA provides a better seal compared with the FM during spontaneous respiration in healthy, awake, bearded subjects. Previously, we showed that the BVGA is more effective and more convenient than the facemask in unconscious anesthetized bearded patients when applied by senior attending anesthesiologists.^14^

### 1.3 Goals of This Investigation

The current study was designed to compare the efficacy and convenience of the BVGA with that of the standard FM in anesthetized bearded patients when applied by anesthesiology trainees with limited experience to simulate closer ventilation by less experienced first responders. The effect of beard length is also assessed.

## 2 Methods

### 2.1 Study Design

**Figure 2:**
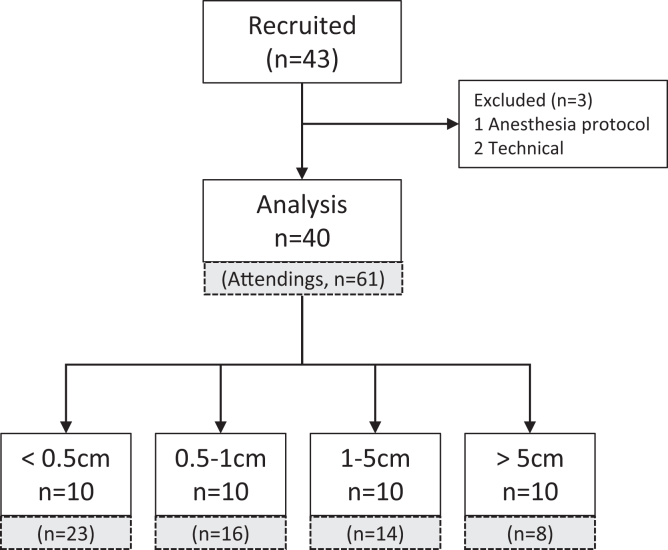
CONSORT study flow. Participant flow and assignment by beard length when administered by trainees (open white boxes). For comparison, lower gray boxes show assignment to beard length in the hands of attending anesthesiologists.^14^

This was a prospective, randomized, single-session, cross-over trial that was approved by the institutional review board, registered prospectively (ClinicalTrials.gov: NCT04376918), and adhered to the applicable Consolidated Standards of Reporting Trials (CONSORT) guidelines. Participants provided written informed consent before entering the study. Bearded patients scheduled to undergo elective surgery with endotracheal intubation facilitated by nondepolarizing muscle relaxants were recruited for this study. Demographics, beard length, and airway-related data were documented. All patients were ventilated by anesthesiology trainees in the early stages of their residency using the BVGA connected to a Guedel airway and an FM with a Guedel airway in situ. Ventilation was provided by each device for 2 minutes. See Figure 2 for a CONSORT diagram of participant flow.

The order of device applications was determined randomly. Vital signs and other specific respiratory parameters were recorded throughout the experiment. At the end of the experimental session, the anesthesiology trainees filled out a questionnaire comparing the BVGA with the FM. Adverse events were documented. The trial ended when the number of participants reached the target sample size. The primary outcome of the study was the expiratory tidal volume (TV), and the secondary outcomes were the end-tidal CO_2_ (EtCO_2_) and user experience. Patients without beards but otherwise similar demographics were recruited as a control group.

### 2.2 Settings and Selection of Subjects

Recruitment took place at the Hadassah Ein-Kerem Medical Center from July 2021 to January 2023. Adult male patients (age > 18 years) with an American Society of Anesthesiology physical status classification score of 1 or 2 and body mass index ≤ 30 who were scheduled to undergo elective surgery were offered to participate in the study if their anesthesia plan included endotracheal intubation and muscle relaxation with a nondepolarizing neuromuscular blocking agent. Exclusion criteria were risk factors for difficult ventilation,^4^ including past or suspected obstructive sleep apnea (per STOP-BANG^15^), maxillo-facial injury, edentulous jaw or unstable teeth, suspected difficult intubation, suspected difficult mask ventilation, or predicted high risk of aspiration.

The patients were recruited after being prepared for surgery in the preoperative area on their way to the operating room. Once informed consent was obtained, the patient received a study code, and all further information was entered on coded sheets. A detailed form, including a standard anesthetic airway assessment and a STOP-BANG questionnaire, was filled, and information from the pre∖filled anesthetic assessment medical record was added. The study team collected demographic information including age (years), weight (kg), and height (cm). Beard length was measured and categorized as follows: <0.5 cm (no beard or stubble), 0.5 to 1.0 cm, 1.1 to 5.0 cm, or >5.0 cm. The sequence of device application (BVGA ⇒ FM or FM ⇒ BVGA) was determined by simple randomization to rule out sequential effects; tickets with the order of interventions were placed in an opaque bag. Prior to the procedure, the trainee drew a single ticket from the bag and performed the interventions accordingly. The ticket was not returned to the bag.

### 2.3 Interventions

The study device BVGA (Fig 1) was developed by the Institute for Research in Military Medicine of the Hebrew University of Jerusalem together with DEA Ltd, Research and Development, Jerusalem, Israel. The device is packaged in a sterile bag and preloaded with the Guedel airway. Once positioned in the patient’s mouth, the lips are aligned within the rail with a finger starting at one end of the corners of the lips and sliding the lips within the rail to the opposite corner of the lips, applying gentle pressure until the device is fully inserted into the mouth and a seal is achieved. This is followed by adjusting the elastic band behind the head and occluding the nose with the clip. Finally, the artificial manual breathing unit bag or ventilation device is connected through the universal adaptor, permitting direct flow of air from the bag valve through the Guedel airway and into the patient. The entire process requires minimal force and dexterity and takes up to 30 seconds, which is comparable to the time required to use standard equipment. The BVGA was placed by the anesthesia residents some of whom participated in the study multiple times. Prior to the study, the residents underwent a 10-minute training session that included an explanation and 5 to 10 placements of the BVGA without a Guedel airway.

### 2.4 Procedures

General anesthesia was induced using midazolam (1-2 mg), fentanyl (2 mcg/kg), lidocaine (1 mg/kg), rocuronium (0.6 mg/kg), and continuous infusion of propofol (2-3 mg/kg). Adequacy of the neuromuscular block was monitored using Neuromuscular Transmission (GE Healthcare), and the depth of anesthesia was monitored with Entropy (GE Healthcare). Prior to the start of the study protocol, the anesthesiologists used the standard FM for ventilatory support. All patients were ventilated using pressure-controlled ventilation with a respiratory rate of 14 breaths per minute, pressure above positive end-expiratory pressure (PEEP) = 14 cm H_2_O, PEEP = 4 cm H_2_O, inspiratory-to-expiratory = 1:2, and FiO_2_ = 1.0.

The study period began when the state entropy was stable and a deep neuromuscular block was established. The anesthesiologist ventilated the patient with both devices (one after the other) – each for 2 minutes. Ventilation was provided using an anesthesia workstation (Carestation 650, GE Healthcare). The physiologic parameters were recorded by live video capturing of the monitor screens (ventilator and hemodynamic monitors). Individual frames were automatically extracted from the digital video files and organized in folders using the “frame extractor” that we developed for this study and can be freely accessed at https://colab.research.google.com/drive/1NMobILUWiHkGWgkejEhydGiN3spGxExH?usp=sharing.

### 2.5 Vital Signs and Ventilation Parameters

Data from the second minute of the 2-minute cycle for each device (14 respiratory cycles) were used for the analysis (Fig 2). Vital signs data included arterial oxygen saturation, systolic, diastolic, and mean arterial blood pressures, as well as heart rate (bpm). Ventilation parameters included EtCO_2_ (mm Hg) and expiratory TV (mL) normalized per kg of predicted body weight. An adequate respiratory cycle was defined as one having either EtCO_2_ > 25 mm Hg or TV > 5 mL/kg. The median value of 14 respiration cycles of TV and EtCO_2_ and the proportions of adequate respiratory cycles delivered were also calculated (%ADQ_TV_ and %ADQ_EtCO__2_, respectively).

### 2.6 Questionnaire

At the end of the procedure, each anesthesiology trainee was given a written questionnaire in which he/she was asked to grade whether the BVGA, compared with the mask, was better, significantly better, the same, worse, or significantly worse pertaining to (1) convenience, (2) physical demand, or (3) tiring (see the form in the Supplementary Material). The percentage of positive scores was calculated.

### 2.7 Data Analysis

The analysis was performed in 2 stages: (1) comparison of the results of ventilation by BVGA vs FM and (2) testing the effect of beard length and level of experience (trainees [current study] vs expert anesthesiologists [previous study^14^]).

#### 2.7.1 Sample size

Sample size calculation for the primary outcome was based on the mean and the SD paired difference in TV between BVGA and FM measured in anesthetized bearded patients from a previous study (1.3 ± 3.3 mL/kg).^14^ Sample size calculations for the subgroup analysis by beard length were based on the EtCO_2_ difference between the BVGA and FM (5.1 ± 4.1 mm Hg).^14^ Using the test for paired means, a sample size of n = 40 for the entire group and n = 6 per beard category (n = 24 for 4 beard length groups) was found to be sufficient to achieve a power of >80% with α = .05. The sample size was determined with PASS-15.0.4 software (NCSS).

#### 2.7.2 Statistics

Intention-to-treat statistics were applied to all patients who underwent the procedure. Data of TV and EtCO_2_ were determined to be normal by the Shapiro-Wilk test, and hence, continuous variables are reported as mean ± SD, and tests are parametric. The effect by order of interventions was tested by 2-tailed unpaired *t* tests (FM first vs BVGA first). Since no sequential influence was found, the data from both groups were pooled (see Supplementary Table S2), and paired *t* tests were used for within-group comparisons (BVGA vs FM). One-way analysis of variance (ANOVA) was used to compare demographic information between beard length categories. The binomial test for single proportions was used to determine if the questionnaire score was significant. Mixed design, 2-way ANOVA was used to test for differences in the proportions of adequate respiratory cycles (assessed from TV or EtCO_2_) when using the BVGA vs the FM with respect to level of expertise or beard length categories. The data from this study were also combined with the data from a previous study^14^ in which the devices were deployed by attending anesthesiologists. The numbers of patients from the previous study are presented in Figure 2. Statistical analyses were performed with SYSTAT, version 13.2 (Systat Software); *P* < .05 was considered significant.

## 3 Results

### 3.1 Characteristics of Study Subjects

Forty-three adult male patients scheduled to undergo general anesthesia with endotracheal intubation facilitated by nondepolarizing muscle relaxants were recruited. Of these, 3 were excluded for technical reasons. Of the 40 included patients (age, 37 ± 17 years; range, 19 to 80 years; body mass index, 25 ± 3 kg/m^2^), 30 had beards, and 10 were without beards or with beards less than 0.5 cm in length (Table 1).

**Table 1:** Demographics by beard length category.

Beard length (cm)	Age (y)	BMI
Total	37.4 ± 16.6	25.2 ± 3.3
<0.5	46.7 ± 16.2	23.8 ± 2.1
0.5-1	26.9 ± 10.1	25.3 ± 2.3
1-5	38.0 ± 13.5	27.3 ± 3.9
>5	38.2 ± 20.7	24.5 ± 3.9

Data are presented as mean ± SD; n = 40; 10 for each beard length category.

BMI, body mass index.

Thirteen anesthesiology trainees performed 40 procedures, of which 6, who were in their first year of residency, performed 29 (73%) of the procedures. These data were compared with those from 61 additional patients of the previous study to whom the experimental procedure was administered by senior (attending) anesthesiologists.^14^

### 3.2 Main Results

**Figure 3:**
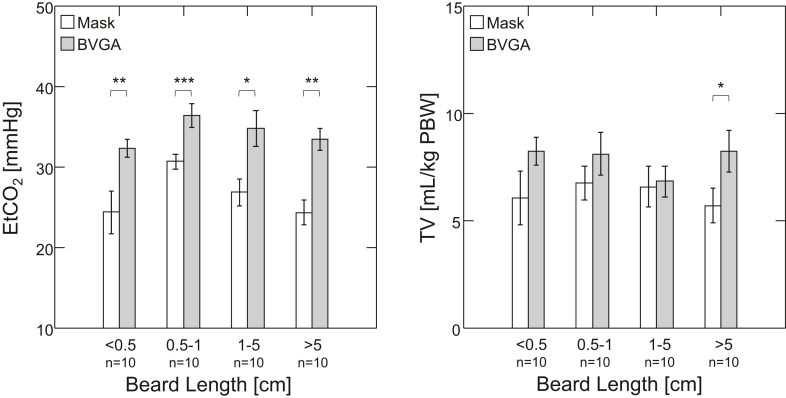
End-tidal CO_2_ (EtCO_2_) and tidal volume (TV) for the Bag-Valve-Guedel Adaptor (BVGA) and face mask (mask) by beard length. Bar graphs represent mean ± SEM. Patients (n = 40) with different beard lengths were ventilated by trainees with both masks and BVGA. Ventilation with the BVGA was found to be superior to the mask by EtCO_2_ across all beard lengths (*P* < .001). TV was improved with the BVGA over the mask only for the longest beard group. ∗*P* < .05; ∗∗*P* < .01; ∗∗∗*P* < .001 by paired *t* test: BVGA vs Mask. PBW, predicted body weight.

Significantly improved respiratory outcomes were found with BVGA compared with those achieved with the traditional FM. This was reflected in higher TV and EtCO_2_ (BVGA vs FM [n = 40]: TV [mL/kg], 7.9 ± 2.5 vs 6.3 ± 2.8, *P* = .003; EtCO_2_ [mm Hg], 34.3 ± 4.9 vs 26.6 ± 5.8, *P* < .001) (Table 2, Fig 3). There was also a significantly greater percentage of adequate respiratory cycles with the BVGA (BVGA vs FM: %ADQ_TV_, 84% ± 31% vs 64% ± 37%; %ADQ_EtCO__2_, 98% ± 7% vs 68% ± 38%, *P* ≤ .001 for both). Regarding the effect of level of expertise, we found that when using the BVGA, the trainees achieved over 97% of adequate respiratory cycles—similar to the level of success attained by expert anesthesiologists (Table 3). Of particular importance, using mixed-design ANOVA (see Supplementary Material for detailed statistical output), the level of expertise was found to be a significant determinant of percent adequate respiratory cycles during ventilation with the FM but not when using the BVGA (Table 3). This emphasizes the important usefulness of the BVGA for less experienced first responders.

**Table 2:** End-tidal CO_2_ and tidal volume for Bag-Valve-Guedel Adaptor vs mask by beard length.

Beard length (cm)	EtCO_2_ (mm Hg)			TV (mL/kg)		
	BVGA	Mask	*P*	BVGA	Mask	*P*
<0.5	32.3 ± 3.3	24.4 ± 7.9	.008	8.2 ± 2.0	6.1 ± 3.8	.133
0.5-1	36.5 ± 4.4	30.7 ± 2.7	<.001	8.1 ± 2.9	6.8 ± 2.4	.088
1-5	34.8 ± 6.6	26.8 ± 4.9	.015	6.8 ± 2.1	6.6 ± 2.8	.780
>5	33.5 ± 4.2	24.3 ± 4.6	.002	8.2 ± 2.9	5.7 ± 2.4	.035

Data are presented as mean ± SD; n = 40; 10 for each beard length category. By paired *t* test: BVGA vs mask.

BVGA, Bag-Valve-Guedel Adaptor; EtCO_2_, end-tidal CO_2_; TV, tidal volume.

**Table 3:** Percent adequate ventilation by experience and presence of a beard.

Beard	All (N = 101)			Residents (n = 40)			Experts^a^ (n = 61)		
	n	BVGA, %	Mask^b^, %	n	BVGA, %	Mask, %	n	BVGA, %	Mask, %
No	33	99 ± 6	88 ± 26	10	97 ± 9	67 ± 40^c^	23	99 ± 3	97 ± 8
Yes	68	99 ± 4	82 ± 31	30	99 ± 5	83 ± 31	38	100 ± 0	82 ± 31

Data are presented as mean ± SD; n = the number of patients.

BVGA, Bag-Valve-Guedel Adaptor.

Mixed design analysis of variance: main effects.

a *P* = .020 experts vs trainee; repeated measures.

b *P* < .001 mask vs BVGA; interactions.

c *P* = .019 with mask, without a beard, trainee vs expert. Note that the BVGA was superior to the face mask regardless of the level of expertise. The detailed statistical output can be found in the Supplementary Material.

Subjective evaluation by the trainees showed that the BVGA was perceived to be more convenient, less physically demanding, and less tiring than the FM in 100%, 96%, and 98% of the cases, respectively (n = 40, *P* < .001 by the binomial test for single proportions).

Vital signs, including heart rate, SPO_2_ arterial oxygen, and blood pressure before the beginning of the experiment and during each intervention, remained within the normal range during ventilation procedures (see Supplementary Table S1 for numerical data). No device-related adverse safety events were encountered.

## 4 Limitations

As per the selection criteria, the participants in this study were of low risk for difficult airway management and were under general anesthesia with neuromuscular blockade during the study. Nonetheless, this study provides important support for testing this approach in prehospital and remote field settings. The duration of the experimental procedure was 2 minutes, which was shorter than what was possibly needed prior to intubation and advanced care. Nonetheless, the BVGA was found to achieve superior respiratory parameters over the FM for the same time interval. The fact that the BVGA was found to be less tiring and required less physical strength points to a major advantage over the FM for situations of single first responder, circumstances requiring longer duration preintubation ventilation, and settings involving responders with limited experience.

## 5 Discussion

Ventilating bearded patients with an FM, whether in the prehospital or hospital setting, is difficult due to less than adequate seal.^4^ The BVGA circumvents this leakage caused by facial hair by enabling a direct, sealed, and universal connection between a manual resuscitator and a Guedel oropharyngeal airway.

The current study showed improved ventilation efficacy when trainees with little experience used the BVGA as opposed to the standard FM. We found the BVGA to achieve superior respiratory parameters than the FM regardless of beard length. The BVGA was also found to be more convenient, less physically demanding, and less tiring than the FM in 96% to 100% of the cases.

The extent of improved efficacy with the BVGA over the FM was greater with the trainees than with the expert attending anesthesiologists, emphasizing the important advantage of this device for the less-experienced caregivers.

Anesthesia trainees have more experience than paramedics or battlefield medics in managing the airway of unconscious patients. This is particularly true following the induction of general anesthesia in the setting of an elective procedure where airway management is done with significant assistance and access to a variety of alternative equipment and techniques. However, paramedics are also trained to manage the airway of injured patients in the field who have variable levels of consciousness and in a variety of challenging settings. This makes the availability of this easily manageable device particularly advantageous for both controlled and less well-controlled settings and for both trained and less experienced caregivers.

Importantly, the BVGA does not significantly differ from standard equipment used for airway management; its application is rather similar to the insertion of a regular oropharyngeal airway, with the exception of the BVGA, which is placed as a seal between the lips.

Our findings indicate that the level of expertise plays a role in the efficacy of FM ventilation, but it does not significantly affect the respiratory outcomes of ventilation performed with the BVGA, although we did not validate this in paramedics. In view of the ease of application and minimal additional training necessary, the next logical step to validate the usefulness of this device will be testing it with emergency medical services clinicians in a prehospital field setting.

The presence of a beard is a well-known risk factor for difficult mask ventilation.^4^, ^5^, ^6^, ^7^ Because it is an intraoral device, the effectiveness of the BVGA was not altered by the presence of a beard, and this was true regardless of the degree of experience of the responder.

The relative increase in TV and EtCO_2_ seen during BVGA ventilation vs the mask is achieved by the direct contact and seal between the bag and the intraoral device that eliminates the potential spaces between the mask and the face created by the beard. Another important contributor to the improved respiratory parameters is the significant reduction in anatomical dead space provided by the BVGA compared with the FM.

In a previous study,^14^ we found that after securing the BVGA, ventilation was possible without hands in 74% of the cases and that it required significantly fewer procedures of chin-lift and jaw thrust compared with when using the FM. The results of this study also suggest that the BVGA can improve early airway management efforts by reducing the manual skills required to achieve the required nose-mouth seal, which is especially challenging in bearded individuals. This is of particular significance in situations of single-responder ventilation.^16^^,^^17^

Supraglottic devices like the LMA are the devices of choice in difficult airway management, both in preintubation ventilation and as an intermediary measure when the intubation is difficult. However, the BVGA intraoral airway is less invasive and is without the other known complications of the supraglottic LMA.^12^ Moreover, according to the Israel Defense Force Medical Corps guidelines, as of February 2024, the use of an LMA is prohibited even by experienced responders in the prehospital setting. Application of a definitive airway is allowed only by certified responders, and others have to use a facemask for ventilation. Thus, the BVGA suggested in this study is a highly desirable alternative for ventilation prior to definitive airway application in prehospital military settings, particularly in bearded subjects and in situations with less experienced responders.

In summary, The BVGA is more effective, more convenient, less physically demanding, and less tiring than the FM for ventilation performed by anesthesia trainees. This device provides superior respiratory parameters than the FM regardless of beard length or in a setting where the use of supraglottic airway devices such as the LMA is limited or prohibited. This study provides important support for testing this approach in prehospital and remote field settings.

## Author Contributions

L.G., S.F., A. Eisenkraft, S.D.G., and R.P. took part in the conception and design of the study as well as in the interpretation of the data and preparation of the initial draft of the manuscript. L.G., S.F., D.F.O.C., A.T., R.A.G.B., A. Erport, J.S., C.M., and R.P. developed different parts of the methodology. D.F.O.C., A.T., R.A.G.B., D.V., A.R., J.S., and C.M. collected the data. A.T. and D.V. validated the data, and A.T. took part in software development. L.G. and S.F. formally analyzed the data and visualized the data together with S.F.O.C. and R.A.G.B. S.D.G., A. Eisenkraft, and R.P. provided resources. S.D.G. and R.P. supervised the experiment and were responsible for the management of the study. All authors revised the data critically for important intellectual content and gave final approval for the version to be submitted. L.G. takes responsibility for the paper as a whole.

## Funding and Support

This study was supported in part by The Stuart Roden Family Research Fund, London, UK; The Saul and Joyce Brandman Fund for Cardiovascular Research; The Alexander Grass Family Research Fund; The Dr Bruce and Baila Waldholtz Research Fund; and The Dr Martin and Grace Rosman Research Fund, Faculty of Medicine, The Hebrew University of Jerusalem, Israel.

## Conflict of Interest

All authors have affirmed they have no conflicts of interest to declare.

Device construction was subcontracted out to DEA Ltd, Research and Development, Jerusalem, Israel, which manufactured it according to the Institute for Research in Military Medicine (IRMM) specifications. DEA Ltd retains the IP. There are not, and have not been, any financial relationships between either the IRMM, The Hebrew University, The Hadassah Medical Organization, or The Israel Ministry of Defense and the subcontractor, DEA Ltd.
