# Supplementary material for: Superiority of the Bag-Valve-Guedel Adaptor Over the Standard Face Mask for Preintubation Ventilation of Bearded Patients by Trainees With Limited Experience: Prospective Controlled Cross-Over Clinical Trial
Source: J Am Coll Emerg Physicians Open. 2025 Jan 13;6(1):100035. doi: 10.1016/j.acepjo.2024.100035 (PMC11852698; doi:10.1016/j.acepjo.2024.100035)
Supplement: Supplementary Material [file mmc1.docx]

# SUPPLEMENTARY INFORMATION

## Vital Signs

Vital signs data included heart rate (HR)[bpm], arterial oxygen saturation (SPO_2_) [%], systolic, diastolic, and mean arterial blood pressure (SBP, DBP, MAP)[mmHg]. Baseline measurements were taken before any intervention when patients were breathing room air spontaneously. The changes in vital signs were consistent with those anticipated following induction of anaesthesia. The patients remained hemodynamically stable throughout the study period.

Supplementary Table S1: Vital signs

| **Variable** | Baseline | Mask | BVGA | p* |
| --- | --- | --- | --- | --- |
| **HR [bpm]** | 72±11 | 72±12 | 72±14 | 0.877 |
| **Sat [%]** | 99±1 | 99±1 | 99±1 | 0.174 |
| **SBP [mmHg]** | 117±20 | 108±19^✝^ | 107±20^✝^ | 0.001 |
| **DBP [mmHg]** | 70±13 | 65±15^✝^ | 63±15^✝^ | 0.004 |
| **MAP [mmHg]** | 86±14 | 79±16^✝^ | 77±16^✝^ | 0.001 |

Data presented as mean±SD; *Repeated measures ANOVA; ^✝^significantly different from baseline by post-hoc test with Bonferroni correction for multiple comparisons. HR = heart rate, Sat = oxygen saturation, DBP = diastolic blood pressure, MAP = mean arterial pressure.

## Normality Test for TV and EtCO_2_

Normal distribution was tested with the Shapiro-Wilk test with p<0.05 considered significant (=not normally distributed). The following Table shows the original statistical output for both primary outcomes by intervention.

Supplementary Table S2: Results of Shapiro-Wilk Test for Normal Distribution

|  | **MASK_TV_IBW** | **MASK_EC** | **BVGA_TV_IBW** | **BVGA_EC** |
| --- | --- | --- | --- | --- |
| N of Cases | 40 | 40 | 40 | 40 |
| Arithmetic Mean | 6.278 | 26.575 | 7.860 | 34.262 |
| Standard Deviation | 2.823 | 5.770 | 2.508 | 4.862 |
| Shapiro-Wilk Statistic | 0.985 | 0.945 | 0.979 | 0.976 |
| Shapiro-Wilk p-Value | 0.866 | 0.051 | 0.663 | 0.554 |

## User Experience Questionnaire

Anesthesiologist ___________________

| Relative to mask, was the BVGA… | | Significantly more | more | the same | less | significantly less |
| --- | --- | --- | --- | --- | --- | --- |
| 1 | Comfortable |  |  |  |  |  |
| 2 | Physically demanding |  |  |  |  |  |
| 3 | Tiring |  |  |  |  |  |

## Testing the Effect of Intervention Order

The difference between Mask and BVGA in TV or EtCO_2_ is presented in the table below. The sequence did not affect either of the variables justifying pooling of the data for further analysis.

Supplementary Table S3: Order of intervention

| Order groups | Begin with mask | Begin with BVGA | p* |
| --- | --- | --- | --- |
| TV [ml**∙kg**^-1^ IBW] | 1.8±3.2 | 1.3±3.3 | 0.617 |
| EtCO_2_ [mmHg] | 7.4±7.0 | 8.0±6.2 | 0.749 |

Data presented as mean±SD of the difference between Mask and BVGA. **comparison between order groups by unpaired t-test; BVGA=Bag Valve Guedel Adaptor. TV = tidal volume, EtCO_2_ = end tidal CO_2_

## Mixed Design ANOVA – Statistical Output

[▼Analysis of Variance](file:///\\Untitled.syo)

Effects coding used for categorical variables in model.

The categorical values encountered during processing are

| **Variables** | **Levels** | |
| --- | --- | --- |
| BEARD (2 levels) | No | Yes |
| EXPERT (2 levels) | No | Yes |

N of Cases Processed: 101

| **Dependent Variable Means** | |
| --- | --- |
| **MASK__ADQ** | **BVGA__ADQ** |
| 84.248 | 99.218 |

| **Repeated Measures Factors and Levels of Dependent Variables** | | |
| --- | --- | --- |
| **Within Factor** | **1** | **2** |
| Intervention | 1.000 | 2.000 |

**Univariate Repeated Measures Analysis**

| **Between Subjects** | | | | | |
| --- | --- | --- | --- | --- | --- |
| **Source** | **SS** | **df** | **Mean Squares** | **F-Ratio** | **p-Value** |
| BEARD | 27.221 | 1 | 27.221 | 0.062 | 0.804 |
| EXPERT | 2,458.096 | 1 | 2,458.096 | 5.570 | 0.020 |
| BEARD*EXPERT | 2,585.248 | 1 | 2,585.248 | 5.858 | 0.017 |
| Error | 42,810.002 | 97 | 441.340 |  |  |

| **Within Subjects** | | | | | | | |
| --- | --- | --- | --- | --- | --- | --- | --- |
| **Source** | **SS** | **df** | **Mean Squares** | **F-Ratio** | **p-Value** | **G-G** | **H-F** |
| Intervention | 10,738.079 | 1 | 10,738.079 | 26.994 | 0.000 | . | . |
| Intervention*BEARD | 4.108 | 1 | 4.108 | 0.010 | 0.919 | . | . |
| Intervention*EXPERT | 1,491.816 | 1 | 1,491.816 | 3.750 | 0.056 | . | . |
| Intervention*BEARD*EXPERT | 2,248.750 | 1 | 2,248.750 | 5.653 | 0.019 | . | . |
| Error | 38,586.437 | 97 | 397.798 |  |  |  |  |

[▼Hypothesis Tests](file:///\\Untitled.syo)**Test for effect called: BEARD*EXPERT**

| **Univariate F-Tests** | | | | | |
| --- | --- | --- | --- | --- | --- |
| **Source** | **Type III SS** | **df** | **Mean Squares** | **F-Ratio** | **p-Value** |
| MASK__ADQ | 4,828.135 | 1 | 4,828.135 | 5.883 | 0.017 |
| Error | 79,605.261 | 97 | 820.673 |  |  |
| BVGA__ADQ | 5.863 | 1 | 5.863 | 0.318 | 0.574 |
| Error | 1,791.178 | 97 | 18.466 |  |  |
